# Cough of Phthisis

**Published:** 1875-04

**Authors:** 


FORMULÆ FOR THE TROUBLESOME COUGH OF PHTHISIS.—

| | |
|---|---|
| ℞—Potassii bromidi, . . . | aa ʒ iss. |
| Potassæ chloratis, . . . . | |
| Ammoniæ muriatis, . . . | |
| Syrup tolutani, . . . . . . | ℥ iv. M. |

Tablespoonful every two or three huurs.

| | |
|---|---|
| ℞.—Tincturæ opii camphoratæ, . . | ℥ i.; |
| Tincturæ belladonnæ, . . . | ʒ i.; |
| Tincturæ hyoscyami, . . . . | ʒ ij.; |
| Spiritus Lavendulæ comp., . . | ʒ i. M. |

Ten drops on a lump of loaf sugar every hour until cough is relieved.—*Charity Hospital, New York.*

